# ERRATUM

**DOI:** 10.1590/0074-0276015011007

**Published:** 2015-11

**Vol. 110 (4): 453-460, 2015.**

**p. 453**

**Interleukin-10 rs2227307 and CXCR2 rs1126579 polymorphisms modulate the predisposition to septic shock**

**should read:**

**Interleukin-10 rs1800896 and CXCR2 rs1126579 polymorphisms modulate the predisposition to septic shock**

**Vol. 110 (6): 797-800, 2015.**

**p. 797**

Financial support: IOC/FIOCRUZ, PAPESIV/VPPDT/FIOCRUZ, FAPERJ-APQ1 (E-26/110.497/2011), CNPq (458858/2014-5)

**should read:**

Financial support: IOC/FIOCRUZ, PAPESIV/VPPDT/FIOCRUZ, FAPERJ-APQ1 (E-26/110.497/2011), CNPq (458858/2014-5), FAPEAM/CNPq/PPP-FAPEAM (010/2011), MCT/CNPq (014/2011)

